# Taxonomy-based data representation for data mining: an example of the magnitude of risk associated with *H. pylori* infection

**DOI:** 10.1186/s13040-021-00271-w

**Published:** 2021-08-28

**Authors:** Inese Polaka, Danute Razuka-Ebela, Jin Young Park, Marcis Leja

**Affiliations:** 1 University of Latvia, Institute of Clinical and Preventive Medicine, Gailezera Street 1, Riga, LV-1079 Latvia; 2 International Agency for Research on Cancer, 150 Cours Albert Thomas, 69372 Lyon, CEDEX 08 France; 3 Center for Gastric Diseases GASTRO, Gailezera Street 1, Riga, LV-1079 Latvia

**Keywords:** Taxonomy, Data representation, Classification, Heterogenous data, Data merging

## Abstract

**Background:**

The amount of available and potentially significant data describing study subjects is ever growing with the introduction and integration of different registries and data banks. The single specific attribute of these data are not always necessary; more often, membership to a specific group (e.g. diet, social ‘bubble’, living area) is enough to build a successful machine learning or data mining model without overfitting it. Therefore, in this article we propose an approach to building taxonomies using clustering to replace detailed data from large heterogenous data sets from different sources, while improving interpretability. We used the GISTAR study data base that holds exhaustive self-assessment questionnaire data to demonstrate this approach in the task of differentiating between *H. pylori* positive and negative study participants, and assessing their potential risk factors. We have compared the results of taxonomy-based classification to the results of classification using raw data.

**Results:**

Evaluation of our approach was carried out using 6 classification algorithms that induce rule-based or tree-based classifiers. The taxonomy-based classification results show no significant loss in information, with similar and up to 2.5% better classification accuracy. Information held by 10 and more attributes can be replaced by one attribute demonstrating membership to a cluster in a hierarchy at a specific cut. The clusters created this way can be easily interpreted by researchers (doctors, epidemiologists) and describe the co-occurring features in the group, which is significant for the specific task.

**Conclusions:**

While there are always features and measurements that must be used in data analysis as they are, the use of taxonomies for the description of study subjects in parallel allows using membership to specific naturally occurring groups and their impact on an outcome. This can decrease the risk of overfitting (picking attributes and values specific to the training set without explaining the underlying conditions), improve the accuracy of the models, and improve privacy protection of study participants by decreasing the amount of specific information used to identify the individual.

**Supplementary Information:**

The online version contains supplementary material available at 10.1186/s13040-021-00271-w.

## Background

Adoption of electronic health record and other electronic registries, which are being merged and analysed for research and healthcare purposes, have created the need for methods that facilitate the use of large heterogenous data sets from different sources, while improving interpretability. Therefore, we propose a data pre-processing approach, which allows us to store the data subsets in a more abstract and compact way – in taxonomies – while the data can still be easily interpreted and understood by researchers, as well as used for data mining and other downstream analyses. The proposed approach uses a clustering algorithm to form taxonomies that describe patient/sample groups in data subsets, and these are then used in data mining applications instead of the raw data set/subsets. Data represented in this format can also be used for statistical analysis, however, it would allow to test hypotheses related to groups and not single factors/attributes.

After this kind of pre-processing a data set can be stored at higher abstraction level, taking less space. Data in this form (data set storing only membership to groups in data subsets, instead of specific characteristics and attribute values) can also be shared more freely because they do not provide specific values for sensitive features and characteristics that could be used to identify an individual, which could breach personal data protection according to the General Data Protection Regulation of the European Union (GDPR) and other legislature protecting personal and sensitive data.

Data at a higher abstraction level minimises the impact of rare and specific differences characteristic to few individuals or samples. These can occasionally lead to overfitting of machine learning models that are becoming an ever more popular means of understanding relationships in data. An overfitted model may assign high importance to small differences of few persons in the group of interest, and misuse them to induce a model, which will fit well to the training data, but will perform poorly in testing and implementation conditions.

Overall, the process of finding groups of similar objects and using membership to these groups is based on the natural manners with which humans behave and the way we are. We all belong to naturally or socially occurring groups (clusters), e.g. we belong to thematic social media ‘bubbles’ based on who we follow or interact with; we all try to eat healthy (or not) and choose which products and foods we eat on a regular basis (vegetarian, low-calorie etc.), etc. Naturally, in any research there will be specific characteristics of a person or sample (factors) that play a significant role and must remain unchanged; however, the secondary descriptions that may have an effect can often be replaced by membership to groups.

In 2001, Kohavi and Provost [1] argued that various fields could benefit from different data representation that includes more knowledge about the domain. Representation of data and features of objects in abstract, often hierarchical forms, is popular in biology, e.g. Gene Ontology and the representation of all organisms in a taxonomic hierarchy, and taxonomy has become a common technique for visualising knowledge and different structures in research and other fields. Domain ontologies are being developed in many fields, and are often applied in data mining tasks, e.g. in recommendation systems [2]. Taxonomies that represent factors of interest are being developed for different tasks in many fields, e.g. diagnosis of chronic pain [3], differentiation of acute myocardial infarction [4], child, youth and family care [5], factors of vaccine uptake [6], description of psychopathology [7], cost-efficiency of care [8], etc.

In most cases the abstract and/or hierarchical data representations are being created manually by field experts. However, not all domains can be efficiently observed and described; therefore approaches for (semi-)automatic generation of data representations are necessary. One of the approaches is to use attribute value taxonomies (AVTs) that hierarchically represent values of a single attribute, which are present in the data (automated generation of AVTs) or which are possible in the domain (human-designed AVTs [9]), creating groups of values that are comprehensible to humans. The most popular approach of automated AVT generation is using hierarchical clustering [10, 11]. These taxonomies also provide attribute value generalisation when cuts are not made at a leaf level, where the original attribute values are stored. Another direction in application of abstract data representation is the use propositionalised attribute taxonomy (PAT), which introduced new representation of AVTs in Boolean attributes, with different values of attributes and their abstractions/groups [12].

There are also approaches that help building taxonomies from unstructured text (e.g. from free-form notes a doctor takes to describe a patient, their symptoms, etc.), learning hierarchical taxonomies based on a semantic approach [13], or concept formation and hierarchical relation learning [14].

However, most of these approaches have been developed for the creation of taxonomies of a single attribute or text. Therefore, in order to build broad meaningful taxonomies, and take advantage of the information and knowledge contained in multi-attribute data sets, we propose the approach to build taxonomies from data subsets consisting of several related attributes, which are then used in downstream analysis and data mining.

## Methods

This study introduces and demonstrates a semi-automated data pre-processing approach that can create interpretable taxonomies, which can be used for classification and data representation at a higher abstraction level without losing information (measured herein by difference in classification accuracy).

A taxonomy in this context is a hierarchical representation of naturally occurring groups of data objects. To use it during classification, a cut in taxonomy must be made, which is implemented as a cut in the hierarchy at a specific level, leaving only the groups of objects that exist at this level.

**Fig. 1:**
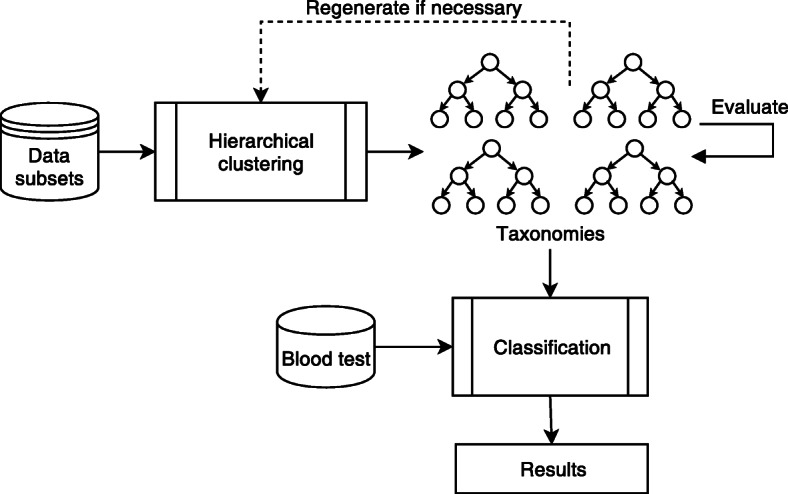
The taxonomy-based classification process

We have investigated the application of hierarchical agglomerative clustering for automated data aggregation into representative groups that can be used to replace descriptive data about each patient or sample. The dendrograms of the clustering process showing hierarchical relationships among groups of people are used as taxonomies. As a result, each patient/sample belongs to a group in each of the subdomains of the full data set (e.g. diet, smoking habits, alcohol consumption, and other features and lifestyle factors) and is now characterised by membership to a described group instead of attribute values. When all the taxonomies have been built, the new descriptions of the patients (memberships to the clusters) can be used in classification or other analysis. The flow-chart of the taxonomy implementation process is depicted in Fig. 1.

The choice of the clustering method, distances and linkage should be made based on heuristics and knowledge of the domain (see Additional file 1 for quantitative metrics and main pitfalls where qualitative assessment should be carried out by a scientist). The hierarchical clustering algorithms in this case are convenient due to an unrestricted number of clusters (as long as the number of clusters is less than the number of cases) and cluster hierarchy trees, which provide additional information, such as the distance of merging of each 2 clusters. This approach also helps to identify outliers, which should be checked and corrected, or often just removed from the analysis. We have used hierarchical agglomerative clustering in which, at the first step, the algorithm merges the 2 closest (similar) records and continues iteratively until all the records belong to a single cluster.

Hierarchies of clusters can then be used in the downstream processes (e.g. classification) instead of a single cut, and the cut can be made during the classification process. Alternatively the cut can be made by the data scientist after analysis of the dendrogram (based on merging distance) and the cluster descriptions (after checking that they are interpretable).

In most cases, Gower’s distance [15] can be used for distance calculations because it is suitable for both continuous and categorical variables. The distance between records *x*_*i*_ and *x*_*j*_ can be computed for each (*k*-th) attribute based on the type of the attribute, as follows:

$$ d\left({x}_i,{x}_j\right)={\sum}_k\frac{w_{ijk}d{\left({x}_i,{x}_j\right)}_k}{w_{ijk}} $$

where *d*_*k*_ is the distance:

for categorical attributes: *d*_*k*_ = 0 if *x*_*ik*_ *= x*_*jk*_ or 1 otherwise.

for continuous attributes: ${d}_k=\frac{\mid {x}_{ik}-{x}_{jk}\mid }{R_k}$, where *R*_*k*_ is the range of the *k*-th attribute.

and the *w*_*ijk*_ is the weight used to process specific cases, e.g. missing and erroneous values.

Other similarity measures were not tested in the application example in this study; however, other distance and similarity measures can be used according to data specifics, e.g. Minkowski (also Euclidean), Pearson distances for continuous data, and Jaccard, Wallace or other similarity measures for categorical data.

When the distance/similarity measures have been set, it is necessary to choose the linkage metric that will define the way the distance between 2 clusters will be measured, and can impact how well different shapes and overlaps of clusters are separated [16]. The most popular approaches are to either choose minimum or maximum distance, calculate the average, or use Ward’s linkage [17]. The minimum distance usually performs poorly when the clusters are not clearly separated, whereas maximum distance breaks clusters apart; therefore average distance or Ward’s distance are usually preferred. The average linkage uses the average distance/similarity of all pairs of records, where one record (*x*_*i*_) belongs to one cluster (*C*_*i*_) with *N*_*Ci*_ records, and the other record (*x*_*j*_) belongs to the other cluster (*C*_*j*_) with *N*_*Cj*_ records:

$$ average=\frac{\sum \limits_{i,j}d\left({x}_i,{x}_j\right)}{N_{Ci}{N}_{Cj}} $$

Instead of using distance between members of different clusters, Ward’s linkage defines the increase of sum-of-squares if 2 clusters are merged and the increase in sum-of-squares is calculated based on centroids m_*Ci*_ and m_*Cj*_:

$$ increase=\frac{N_{Ci}{N}_{Cj}}{N_{Ci}+{N}_{Cj}}{\left\Vert {m}_{Ci}-{m}_{Cj}\right\Vert}^2 $$

In order to create compact clusters, the minimum increase is chosen at each step to minimise the total within-cluster variance and the subsequent inter-cluster distance after the merger.

Therefore, to create taxonomies for each data subset the following steps must be followed:

1. Remove outliers (e.g. remove values that are more than 3 standard deviations from the mean or using other measure based on data specifics).
2. Remove or impute the missing data and normalise the continuous data (e.g. using Z-score or some other approach that would not create values that affect the distances).
3. Calculate a distance matrix using, e.g., Gower’s distance.
4. Cluster the records using hierarchical agglomerative clustering.
5. Analyse the dendrogram and the sizes of clusters at top N levels (usually 10 levels are sufficient when the data is not very complex), as well as cluster characteristics (attribute values common to clusters).
6. Replace the subset with the taxonomy, or make several cuts for feature selection.

**Fig. 2:**
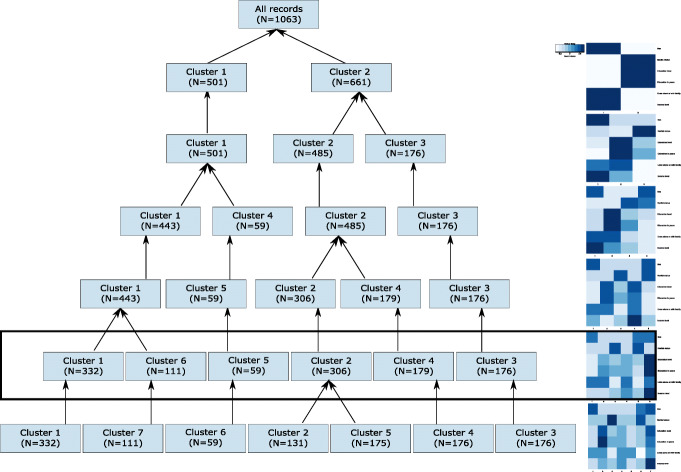
Cluster merges in the last 7 levels of agglomerative clustering using sociodemographic factor data subset

The taxonomy for the sociodemographic data and the cut at 6 clusters is shown in Fig. 2. The figure illustrates the top levels of dendrogram and the characteristics of all clusters in the corresponding level of the dendrogram (mean value of sex, marital status, education level, education in years, if the person lives alone (coded as lower value, therefore lighter blue in the heatmaps) or with family, and income level).

**Fig. 3:**
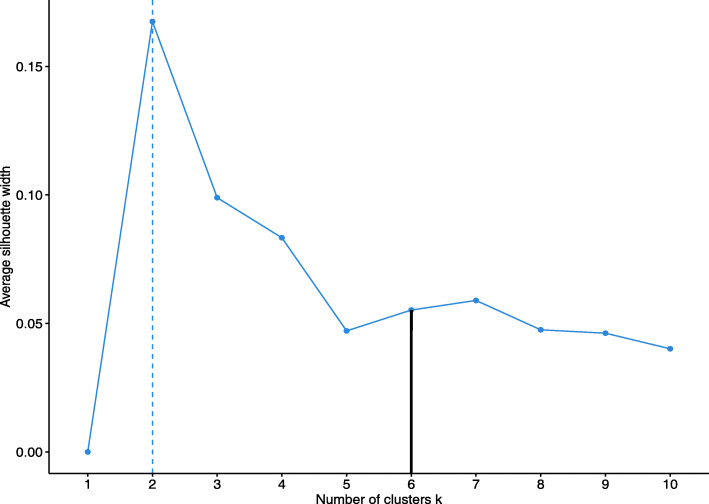
Silhouette width for 10 clusters in sociodemographic factor data subset

The level that should be used for the cut, can be chosen based on the descriptive statistics values of clusters, or using a cluster evaluation metric like Silhouette width (Fig. 3). In many cases the evaluation metrics may favour cluster sets (all clusters at one level) that are not the most informative ones. For example, the Silhouette width in the sociodemographic data subset points to a cut at 2 clusters. However, in Fig. 2, we can see that both clusters are not very informative. They are split to show two very different groups of people that are consistent with the statistics of Latvia: women, who have higher education (gender gap in tertiary education attainment), are living alone (life expectancy for men is 70 years, while for women it is 80) and have lower income; and men, who have lower education level, live with their families and have higher income. Therefore, more clusters are necessary to show the dynamics of subgroups, and the chosen level for taxonomy cut is at 6 clusters.

**Fig. 4:**
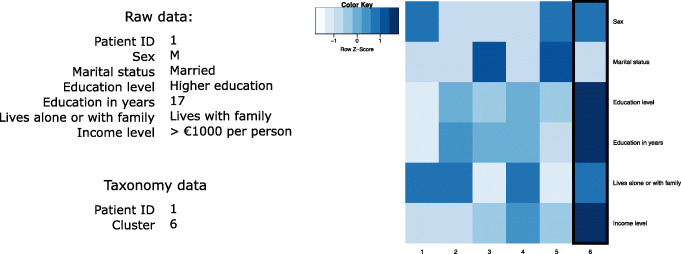
Example of one record: raw data and the corresponding cluster

The membership to these 6 clusters is then encoded into a single attribute and used as input for classification instead of the separate attributes describing these sociodemographic data, also keeping a key that describes each cluster at that cut (see Fig. 4). An example of data transformation for one record is given on the left side of Fig. 4. The key for cluster 6 is: sex - male, marital status – married, education level – higher education, education in years – 16.4, lives alone or with family – with family, income level – more than €500.

If the downstream analysis of the data includes attribute selection or a classification algorithm which includes attribute selection, several cuts can be used as separate attributes to select the most informative cut downstream.

Evaluation of the applicability of the taxonomy was carried out using popular classification algorithms that include feature selection, such as rule-based classification methods (FURIA [18], RIPPER [19], RiDoR [20]) and decision tree based classification methods (C4.5 [21], CART [22], Random Forest [23]). The algorithms were selected due to the built-in processes to choose features (and therefore the best cut in the taxonomy) and transparent models that can be easily interpreted by experts. The classification was carried out using Weka [24] Experimenter environment. Each evaluation included running the classification algorithm with the initial (raw) data set and the clustered data set, which used information representation at higher abstraction level using taxonomies. The classification was executed using 10-fold cross-validation for 100 runs with each data set (100 different randomisations of the data sets). The results were evaluated based on the following metrics:

- Change in the overall accuracy (records classified correctly as a percentage of the total number of records in the data set) and area under the ROC curve, which show whether the use of higher abstraction leads to significant loss of information necessary to discriminate between the classes.
- Change in the true positive rate (TPR; part of records classified as positive that are actually positive) and true negative rate (TNR; part of records classified as negative that are actually negative), which show if there is loss in information that has an impact on one or the other class.
- Number of rules or size of the decision tree (number of nodes in the classifier), which demonstrates the ability of the algorithms to use the information held in the attributes. There is a risk that the classifiers can be built using one or no attributes (the attributes cannot be used by the selected simple algorithms to separate the classes well enough) and are therefore useless, or that the classifiers can be very large, which suggests overfitting.

To evaluate the differences between the raw and the processed data, each metric for the pair of data sets and each classification method was analysed using the Mann-Whitney U test. To evaluate the usability of the clusters (their interpretability and significance as risk factors), some of the most meaningful taxonomies and clusters were assessed as risk factors (pointing to *H. pylori* positivity) using logistic regression, OR and its statistical significance.

### Description of the data

The proposed approach was tested using the example of *H. pylori* (*H. pylori*) infection to assess the change in informativeness in discriminating between positive and negative cases. This topic was selected because of the role of certain parameters, including socioeconomic factors, hygiene, education and lifestyle, in the acquisition of the infection. The topic has also been extensively studied, providing a source for comparison of the results.

We have analysed the association between *H. pylori* seropositivity and a variety of individual factors in the same cohort in previous studies. However, the large number of factors available for the analysis (questionnaire data for more than 100 questions), possible confounding factors in the data, and interrelation among sociodemographic, lifestyle and diet habit variables may have limited the possibility to identify specific factors associated with the infection [25]. Using taxonomies could mitigate these limitations. The most common cooccurring values would be clustered into one group revealing the interrelations; associations with other factors or groups of factors that often occur together can then be discovered more easily.

This approach was evaluated on a large data base from the GISTAR study in Latvia [26] to gain more insight into factors that can affect the presence of *H. pylori* in the stomach. The GISTAR study database was built as a data repository for a randomised population study of *H. pylori* eradication and pepsinogen testing for the prevention of gastric cancer mortality. This initiative is important because *H. pylori* had been classified as a group I carcinogen by the International Agency for Research on Cancer of the World Health Organization (WHO) in 1994 [27], and is common among the Latvian population [28]. Therefore, it is necessary to diagnose and monitor the positive cases, as well as identifying risk factors that may contribute to the presence of *H. pylori* infection. From this database, we selected 1082 patients from the intervention group who had undergone gastroscopy (Giemsa staining *H. pylori* histology test used as the golden standard to determine presence of *H. pylori* infection). The following self-reported information subsets were extracted from the database:

- Sociodemographic factors: 7 attributes, including sex, age, marital status, education, living arrangements, monthly income level per household member after tax.
- Employment history: 9 attributes describing employment – type of job and length of employment in years.
- Hazards and harmful factors: 10 attributes describing contact with heavy metals, asbestos, radiation, etc.
- Diets: 9 attributes describing the diet, e.g. vegetarian, vegan, gluten-free etc.
- Food preferences: 38 variables describing overall preferences, consumption frequency of vegetables, bread, fruit, dairy products, other food of animal origin, drinks etc., divided into subgroups of food origin and overall preferences.
- Alcohol consumption: 11 attributes describing g of alcohol per month consumed with different types of alcoholic beverages (beer, cider, wine, string spirits etc.).
- Smoking: 7 attributes describing previous and ongoing smoking habits.

These data are used in this study to build taxonomies and then classify patients into *H. pylori* positive and negative groups using the proposed data representation approach, taxonomy-based classification and the results of *H. pylori* Giemsa staining tests during histology as the golden standard (target attribute). *H. pylori* antibody test results were added as a single additional factor (*LZ Helicobacter Pylori Antibody Test Kit*, Eiken Chemical Co., Ltd., Tokyo, Japan; 21 patients who had antibodies measured with other tests or did not have the test result were excluded from the classification step in order to exclude additional factor that might have impact on the results). This *H. pylori* serology test is 84% accurate compared to histology in the patient group selected this study. The descriptive characteristics of the patient group used in the evaluation are given in Table 1.

**Table 1:** Descriptive values of the data set

Attribute	Value	*H. pylori* histology Positive (*N* = 609)	*H. pylori* histology Negative (*N* = 473)	All (*N* = 1082)	*p*-value
Age (years)		52.10 ± 6.66	52.01 ± 6.65	52.06 ± 6.66	0.887**
Sex	Female	326 (53.5%)	286 (60.5%)	612 (56.6%)	0.022*
Education level (graduated)	N/A	1 (0.2%)	0 (0.0%)	1 (0.1%)	0.130*
	Secondary school	22 (3.6%)	14 (3.0%)	36 (3.3%)	
	High school	99 (16.3%)	76 (16.1%)	175 (16.2%)	
	Vocational school	297 (48.8%)	202 (42.7%)	499 (46.1%)	
	Higher education (college/ university)	190 (31.2%)	181 (38.3%)	371 (34.3%)	
Income level	Don’t know	25 (4.1%)	17 (3.6%)	42 (3.9%)	0.876*
	< 100€	30 (4.9%)	17 (3.6%)	47 (4.3%)	
	100€-250€	171 (28.1%)	124 (26.2%)	295 (27.3%)	
	250€-500€	274 (45.0%)	222 (46.9%)	496 (45.8%)	
	500€-1000€	80 (13.1%)	66 (14.0%)	146 (13.5%)	
	> 1000€	5 (0.8%)	5 (1.1%)	10 (0.9%)	
	Will not answer	24 (3.9%)	22 (4.7%)	46 (4.3%)	
Has smoked at least 100 cigarettes	No	326 (53.5%)	289 (61.1%)	615 (56.8%)	0.043*
	Yes	282 (46.3%)	183 (38.7%)	465 (43.0%)	
	N/A	1 (0.2%)	1 (0.2%)	2 (0.2%)	
Alcohol consumption per month (ethanol, g)		131.2 ± 197.4	105.5 ± 175.7	112.0 ± 188.6	0.004**
*H. pylori* serology	Positive	570 (93.6%)	137 (29.0%)	707 (65.3%)	< 0.001*
Total		609	473	1082	–

*Chi-square test

**Mann-Whitney U-test (the attribute is not normally distributed)

## Results

**Fig. 5:**
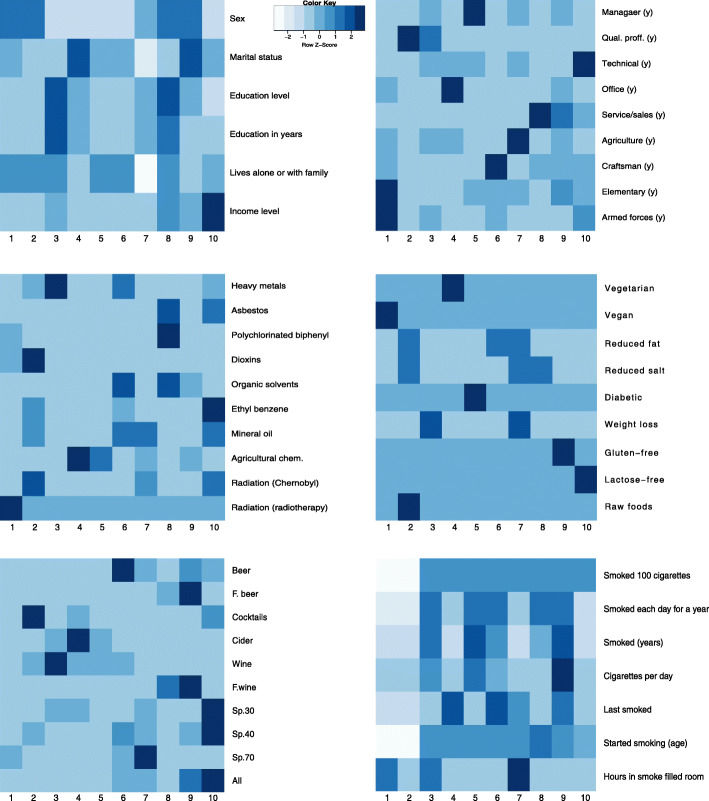
Heatmaps of clusters in subsets: sociodemographic factors, employment history, exposure to potentially hazardous materials, diets, alcohol consumption, smoking

The previously described data subsets were pre-processed according to the required steps: continuous variables were normalised using Z-score and records with outliers (more than 3 standard deviations from the mean) in the alcohol consumption subset (obvious outliers due to human error or lack of comprehension of questions were excluded (e.g. consumption of several litres of alcohol per sitting). The subsets were then clustered using hierarchical agglomerative clustering with Gower’s distance and Ward linkage. The analysis shows distinctive and descriptive clusters at cuts of 10 or less, therefore taxonomies that were used at later analysis stage did not consist of more than 10 levels. Heatmaps describing clusters in some of the subsets are given in Fig. 5. The presented clusters show that there are usually one of two patterns: either one (or few) dominant feature specific only to one cluster (e.g. employment history in the top right heatmap; a person usually spends most of their working life doing similar jobs) or clusters described by different levels of the same factors (e.g. sociodemographic factors in the top left heatmap; cooccurrence of higher education and income etc.).

After clustering of the subsets of the approbation data set, membership of each record to a cluster was saved at different levels to preserve the hierarchy for later cuts during classification. The clustered data set was used for classification using methods that incorporate feature selection (and therefore cuts in taxonomies can be made at this step of classifier induction), which included rule induction methods FURIA, RIPPER, RiDoR and decision tree induction methods C4.5, CART and Random Forest. The average results of 100 runs, in which each included 10-fold cross-validation to build and test a model, are presented in Table 2.

**Table 2:** Classification results (*H. pylori* positive vs negative) in clustered and raw data

	Data set	Area under ROC (95% CI)	Percent correct (95% CI)	True positive rate (95% CI)	True negative rate (95% CI)	Number of rules (95% CI)	Tree size (95% CI)	Serialized model size in bytes (95%CI)
FURIA	Clustered	0.871^a^ (0.87…0.873)	87.3 (87.1…87.5)	0.799^a^ (0.795…0.802)	0.930^a^ (0.928…0.932)	9.5^a^ (9.2…9.7)		592230^a^ (588,461…595,998)
	Raw	0.880^a^ (0.878…0.882)	86.9 (86.7…87.1)	0.824^a^ (0.821…0.828)	0.904^a^ (0.902…0.907)	11.6^a^ (11.2…11.9)		976093^a^ (970,497…981,689)
RIPPER	Clustered	0.872^a^ (0.87…0.874)	87.1 (86.9…87.3)	0.804^a^ (0.801…0.807)	0.923^a^ (0.921…0.925)	4.8^a^ (4.7…4.8)		18746^a^ (18,706…18,785)
	Raw	0.881^a^ (0.879…0.883)	86.9 (86.7…87.2)	0.833^a^ (0.83…0.837)	0.897^a^ (0.895…0.9)	4.2^a^ (4.2…4.3)		25909^a^ (25,882…25,936)
RIDOR	Clustered	0.847 (0.845…0.849)	85.8 (85.6…86.0)	0.760^a^ (0.756…0.765)	0.934^a^ (0.931…0.936)	7.3^a^ (7.2…7.5)		7940^a^ (7796…8083)
	Raw	0.85 (0.847…0.852)	85.6 (85.4…85.8)	0.802^a^ (0.797…0.807)	0.897^a^ (0.894…0.9)	6.4^a^ (6.3…6.4)		5412^a^ (5328…5495)
C4.5	Clustered	0.891^a^ (0.889…0.894)	86.9^a^ (86.7…87.1)	0.802^a^ (0.798…0.806)	0.921^a^ (0.918…0.923)	23.3^a^ (22.8…23.8)	28.6^a^ (28.0…29.1)	17914^a^ (17,819…18,010)
	Raw	0.868^a^ (0.865…0.87)	86.1^a^ (85.9…86.3)	0.826^a^ (0.822…0.829)	0.889^a^ (0.886…0.891)	26.3^a^ (25.5…27.1)	37.3^a^ (36.3…38.4)	25801^a^ (25,608…25,994)
CART	Clustered	0.867^a^ (0.865…0.869)	86.1^a^ (85.9…86.3)	0.786^a^ (0.783…0.79)	0.919^a^ (0.917…0.921)		6.3^a^ (6.2…6.5)	603873^a^ (603,126…604,620)
	Raw	0.889^a^ (0.887…0.891)	87.8^a^ (87.6…88.0)	0.845^a^ (0.842…0.848)	0.904^a^ (0.901…0.906)		5.1^a^ (5.1…5.2)	909785^a^ (908,345…911,226)
Random Forest	Clustered	0.887^a^ (0.885…0.889)	82.2^a^ (82.0…82.4)	0.736^a^ (0.732…0.74)	0.888^a^ (0.886…0.891)			4537297^a^ (4,532,554…4,542,040)
	Raw	0.915^a^ (0.913…0.916)	85.2^a^ (85.0…85.4)	0.754^a^ (0.75…0.758)	0.927^a^ (0.925…0.929)			4003216^a^ (3,999,312…4,007,121)

^a^statistically significant difference (*p* < 0.05, Mann-Whitney U test)

The table shows that the AUC values in the data sets with taxonomies are usually slightly lower, which could be due to some loss of information, but the differences are small and the AUC values remain high, so this should not be a concern for future use. On average, the use of taxonomies slightly improves the overall accuracy. The difference is significant for 3 methods; for C4.5 it increases, whereas for CART and Random Forest it slightly decreases. The decrease in accuracy of CART algorithm could be due to its specifics; it creates binary splits that are not as useful for taxonomies as for binary or nominal attributes. The results are also similar for TPR and TNR, although the data set with taxonomies shows a slightly higher TNR. Most differences are within 1–2%, which means that there is no significant loss of information when applying higher abstraction of data representation.

There are significant differences regarding the sizes of the models, especially in C4.5. The models built with the use of taxonomies are slightly larger when the initial data are very small (RIPPER, RIDOR, CART). They often use only the serology variable when rules and trees are induced from the initial data, which did not increase the testing accuracy or explain the reasons for positivity. In the case of C4.5, the situation is reversed; the models built on the initial data set are larger (the average tree size is 37.4 nodes) but are on average 2.3% less accurate than the smaller trees built using taxonomies. The average serial model size (in bytes) is smaller for the models built with taxonomies in 4 cases out of 6. This suggests that the use of taxonomies could lead to more informative models while keeping the risk of overfitting low.

Whereas taxonomy-based classification models are sometimes smaller due to using membership of a record to a cluster instead of values of attributes, the description of clusters can be successfully used to interpret the results. In the given example evaluation, the most significant examples of the taxonomies that help in differentiating between *H. pylori* positive and negative patients include food/diet, occupation, environmental hazards and sociodemographic factor taxonomies.

**Fig. 6:**
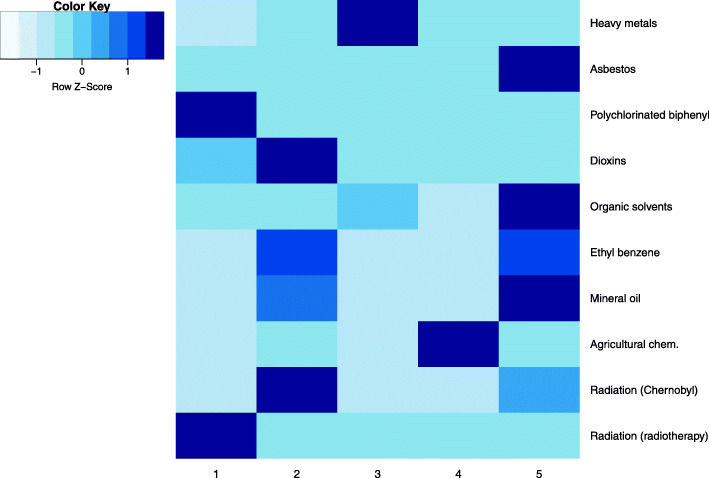
Taxonomy: exposure to hazards, 5 groups; darker colour represents longer exposure (in years)

In the taxonomy that holds information about the exposure to different hazards (and the period of exposure), 73.2% of people exposed to agricultural chemicals were *H. pylori* positive (Fig. 6); this group included 6.7% of the study individuals. The difference among the identified hazard groups is statistically significant (Chi-test *p* = 0.045), and OR of *H. pylori* positivity in the group exposed to agricultural chemicals was 2.09 (95%CI: 1.21…3.62, controlling for age, sex and alcohol consumption) compared to the group that had some prior exposure to polychlorinated biphenyl (transformers, light bulbs, reagents) or radiotherapy (was the largest group in the study population). Analysis of single factors (raw data) shows that longer exposure (> 6 years) to agricultural chemicals is linked to a higher infection risk (OR = 2.24, 95%CI: 1.22…4.10, compared to no exposure, *p* = 0.009), whereas a shorter exposure period is not statistically significant.

**Fig. 7:**
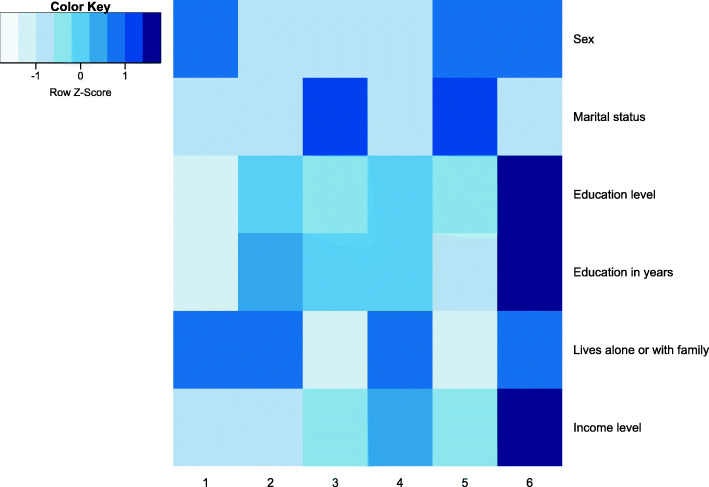
Taxonomy: sociodemographic factors, 6 groups; darker colour represents majority of males, cohabiting/married people, higher level

The difference among groups in the sociodemographic taxonomy at 6 clusters (Fig. 7) was not significant (Chi-square *p* = 0.086). However, the taxonomy holds a cluster (number 4), which includes a group of participants in which 50.3% were negative, whereas negative cases in the entire study population were only 43.5%. The odds ratio of being positive compared to cluster 3, which is also predominantly female but is characterised by lower income and lower education level, is 0.76 (95%CI: 0.48…1.20), when controlling for age, sex and alcohol consumption. Another interesting cluster is cluster 6, which represents well educated men with high income. The OR for members of this cluster to be positive when compared to cluster 1 (less educated men with lower income) is 0.647 (95%CI: 0.42…1.01; *p* = 0.056). Although this difference is insignificant, it could improve with a larger cohort because only 155 individuals belong to cluster 4 and 108 belong to cluster 6. Analysis of separate factors (raw data) showed no significant results (*p* = 0.618 for the binary logistic model, including all of the frequently represented attributes in the cluster).

**Fig. 8:**
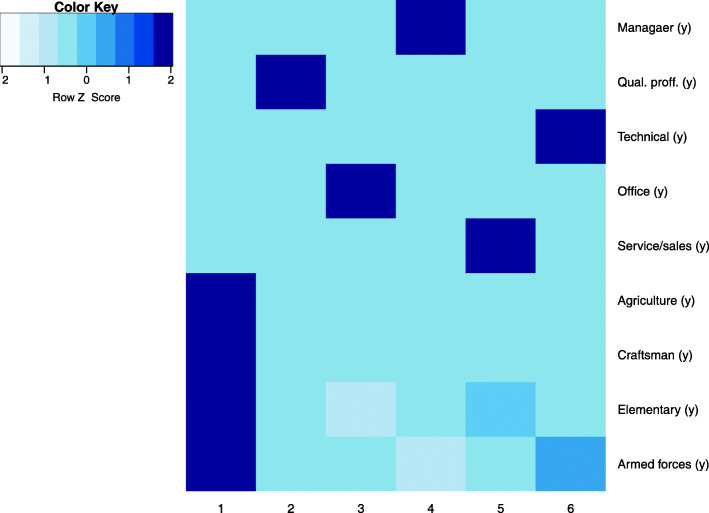
Taxonomy: employment history, 6 groups; darker colour represents longer employment (in years)

The taxonomy that represents employment history (occupational groups and length of occupation, Fig. 8) also defines several groups that have a higher *H. pylori* positivity rate - a group of professions that generally require less education (agriculture, craftsmanship, armed forces and elementary professions, as well as service and sales). In these groups, positivity rate is > 60% (61.5% for the first cluster, 59.3% for the fifth cluster). The difference among the groups is not significant (Chi-square *p* = 0.072), but clusters 1, 2, 3 and 4 are significant factors in a binomial regression. The odds ratios of other clusters, taking cluster 1 (longer period of time in jobs with lower pay, and possibly reduced access to hygiene facilities) as reference, are OR = 0.687 (95%CI: 0.491…0.963) for qualified professionals (*p* = 0.029), OR = 0.589 (95%CI: 0.349…0.995) for office workers (*p* = 0.048), and OR = 0.626 (95%CI: 0.398…0.985) for managers (*p* = 0.043). However, controlling for age, sex and alcohol consumption, reduces the significance of these factors to *p* = 0.082, *p* = 0.136 and *p* = 0.051 respectively. Clusters 2 and 3 are predominantly female (71.5 and 93.9% respectively), suggesting potential influence of this factor. The binomial regression does not show the years worked in any of the professions as a statistically significant factor when the professions are analysed as separate attributes.

**Fig. 9:**
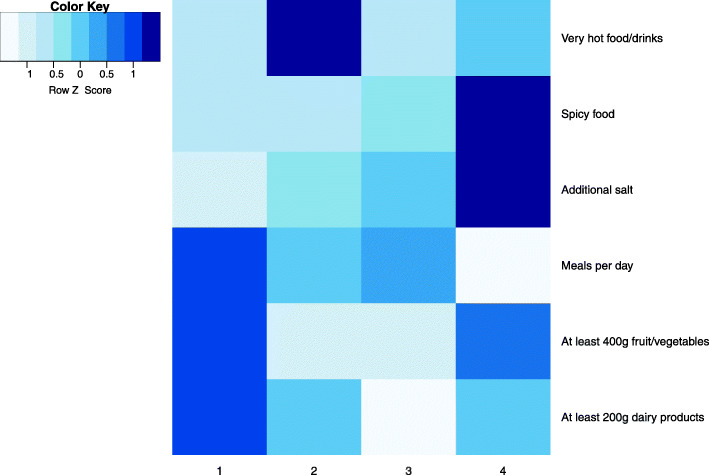
Taxonomy: eating habits, 4 groups; darker colours represent higher percentage of ‘Yes’, more frequent use (salt) and more meals per day

Another important taxonomy represents eating habits (Fig. 9). The group of participants who enjoy spicy food and add additional salt to their food have a 63% positivity rate for *H.pylori;* this group includes 25.7% of the individuals (cluster 4). The difference among these groups is significant (Chi-square *p* = 0.033) and the 4th group has higher *H.pylori* positivity (OR = 1.40, 95%CI: 1.01…1.95, *p* = 0.043 compared to the first group, which is characterized by participants who do not prefer hot, spicy or salty foods, eat frequently and consume more fruit, vegetables and dairy products, controlling for age, sex and alcohol consumption). When separate attributes are analysed in the raw data, only eating spicy food comes close to being a significant factor (OR = 1.37, 95%CI: 1.04…1.80, *p* = 0.06) but is also rendered insignificant when controlling for age, sex and alcohol consumption, while the model holding all of the attributes has borderline significance (*p* = 0.052). There are some interrelations with sociodemographic groups - the cluster with a lower education level (No. 1 in Fig. 8) had significantly more people belonging to the 3rd and 4th clusters of eating habits (Fig. 9), i.e. those who consumed little vegetables/fruit and dairy products, and those who often consumed spicy/hot foods and added extra salt to their meals. The cluster with higher education and income level more often belonged to cluster 1 of food preferences (eating more frequently, consuming at least 400 g fruit/vegetables daily, at least 200 g dairy products daily, but not hot/spicy foods or adding extra salt to their meals).

**Fig. 10:**
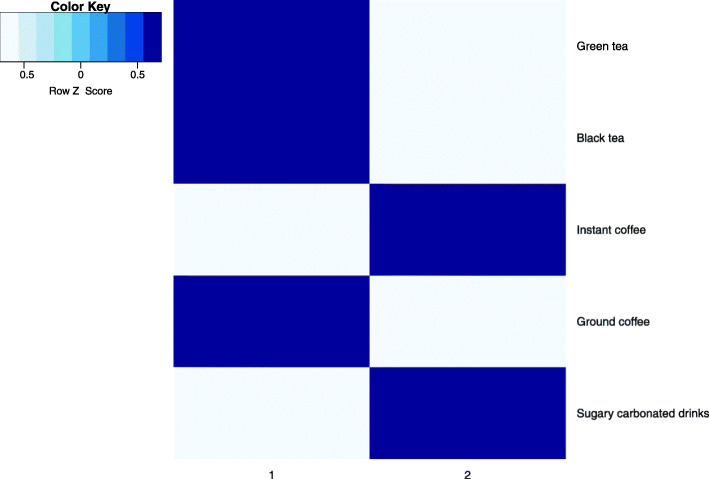
Taxonomy: preferred beverages, 2 groups; darker colour represents more frequent use

Taxonomy of beverage consumption at 2 cluster level (Fig. 10) shows that cluster 1 (frequent consumption of green and black tea, and ground coffee) has less *H. pylori* positive individuals (54.2%), whereas cluster 2 (frequent consumption of instant coffee and sugary soft drinks) has more positive cases (63.8%), a significant difference (Chi-square test *p* = 0.009). The odds of being *H. pylori* positive are almost half as high in cluster 2 (OR = 1.52, 95%CI: 1.12…2.08, *p* = 0.008, controlling for age, sex and alcohol consumption). The separate attributes are not significant factors, nor is the binary logistic regression model including these attributes.

## Discussion

We have presented an approach for data representation that is based on the use of a higher abstraction of the description of patients or other study subjects (based on taxonomies and cuts in the taxonomies) instead of using the actual values of measurements or characteristics. This approach is based on the natural co-occurrence of factors that affect an outcome and allows for a more abstract description of a study subject, whilst preserving the interpretation of the results of analysis and the data-mining models induced from these data.

While there might be some considerations for not using taxonomies instead of specific attributes (e.g., the impact of one or a few specific factors and necessity for statistical analysis that is based on one-attribute factors), the use of taxonomies for additional descriptive attributes (e.g. data subsets from different sources that can be interpreted as descriptions of groups) is useful. The loss of information encountered in the evaluation study is small, which is supported by classification results. The decrease in accuracy metrics was small (±2%, often less), and in the case of overall accuracy, the results of taxonomy-based classification were often better.

The most important step in this taxonomy-based data analysis is taxonomy creation. This study presents taxonomy development using Gower’s distance and Ward’s linkage due to the size and interpretability of the clusters produced by these methods. The size of the clusters created is more similar and there are fewer occasions where clusters consist of a few very specific cases. Nevertheless, the shape of the clusters, as well as the distribution of attribute values, may have significant influence. For example, attributes with exponential distribution that have few records that deviate from 0 (or the group close to 0, e.g. alcohol consumption) leave few cases to be clustered outside the zero consumption cluster. Interrelation between several factors is often observed in different fields, i.e. in healthcare. For example, instead of using each product/habit separately, the participants are divided into groups characterised by diet/lifestyle specifics that include several products and/or habits. The evaluation of this approach was carried out on the data of GISTAR participants who had undergone endoscopy and had been tested for *H. pylori*. This topic was chosen because factors affecting the risk of infection have been widely studied. Previous studies in the field have used specific individual factors instead of our proposed taxonomies. This evaluation allowed us to identify significant clusters (descriptions of more positive or more negative groups), compare their impact to the results obtained using single-attribute factors, and compare our findings to other studies, hence assessing the utility of taxonomies.

We have previously found associations between *H. pylori* seropositivity and several factors when analysing individual factors within the GISTAR project. We compare and discuss our main findings in the evaluation analysis in relation to previous studies below to demonstrate the similarities and differences between analytic approaches and the interpretability of taxonomy-based results.

Interrelation between several factors was observed in our analysis. Although individuals with higher education and income, as well as those working higher tier jobs were less likely to have *H. pylori*, these associations were rendered statistically insignificant after controlling for gender, age and alcohol. Similarly, in our previous study in which taxonomies were not used, sensitivity analyses showed that both education and income were interrelated and associated with other factors directly associated with *H. pylori* [25], but associations became insignificant in multivariate analysis, althoughother studies reported that individuals with higher education and income are less likely to be infected with *H. pylori* [29, 30]. However, with taxonomies, unlike in our previous study, we were able to identify subgroup based trends for income and education when stratifying by sex. Given the small size of these particular clusters, further analysis with a larger study population paying particular attention to gender based disparities is necessary.

On the other hand, in the diet taxonomy, specific dietary habits were significantly associated with *H. pylori*, even after controlling for age, sex and alcohol. In the diet taxonomy, participants that consumed more spicy food and added additional salt to their meals had a higher *H. pylori* positivity rate (OR = 1.49, *p* = 0.015) than those who did not frequently consume hot, spicy nor salted foods, ate more frequently, and consumed more fruit, vegetables and dairy products. Other studies show that high consumption of salt increases the risk of *H. pylori* infection [31] with spicy food also mentioned as a possible risk factor [32].

The odds of being *H. pylori* positive are almost half as high in the cluster with individuals that prefer soft drinks and instant coffee (OR = 1.55, *p* = 0.005) compared to those consuming more tea and ground coffee. This is also supported by other studies where tea consumption has been linked to reduced *H. pylori* positivity [33, 34], whereas soft drinks are reported to have the opposite effect [35].

We previously found associations between *H. pylori* and 4 of the dietary factors mentioned above in univariate analysis, with 3 of them remaining statistically significant in multivariate analysis (consumption of very hot food/drinks, ≥200 g dairy daily, ≥400 g vegetables/fruit daily) [25]. Most studies on *H. pylori* and associated diseases (gastric atrophy, gastric cancer) have focused on specific foods. Epidemiologic reviews have suggested that when examining diet and disease in the context for public health, the best approach is analysing food groups and patterns instead of isolated foods and nutrients [36, 37]. In our example, we could identify associations that we could not previously determine without the use of taxonomies, e.g. associations with exposure to hazardous substances, employment history, preference of spicy food and adding extra salt, as well as consumption of some beverages. This can be explained by the use of more attributes in the same analysis, as well as the use of co-occurring and related attributes together.

We had also suggested that dietary habits could be indirect markers of socioeconomic status [25], a hypothesis that our present analysis using taxonomies seems to support. Cluster analysis showed that participants with a lower level of education tended to consume less vegetables, fruit, and dairy products, but more spicy and hot foods, as well as add extra salt to their meals. The cluster with higher education and higher income level were more likely to belong to the cluster that consumed more vegetables, fruit and dairy, but did not consume spicy and hot foods, nor add extra salt to their meals. These interrelations fit those observed for the *H. pylori* positive cluster.

Analysis suggests that beverage clusters (soft drinks and instant coffee vs. tea and ground coffee) might also be linked to income level, and to the attention participants pay to a healthy diet and lifestyle. Although most of the tea and ground coffee consumers were in the high education/income cluster (86% of the participants in the cluster), a significant difference was observed in the 5th sociodemographic cluster (lower education and lower income, mostly males living alone), where 30.8% of the participants consumed mostly sweetened carbonated drinks and instant coffee.

By using our approach, more factors were found to be significantly related to *H. pylori* infection, and although income and education were not significantly directly linked to the infection, subgroup analysis showed possible interrelation. This can be partially explained by lower granularity of the data in taxonomies. Using clusters, it was possible to examine further the role of sociodemographic factors, linking them to the dietary habits directly associated with *H. pylori* seropositivity. Yet the findings of our current study confirm our previous study - dietary factors seem to be the most important associated with the presence of *H. pylori* infection.

While there can be limitations to the application and researchers choose to use some factors as separate attributes, we consider taxonomy-based representation and analysis as an accurate and interpretable alternative, especially in cases with large data sets pooled from different sources. The proposed approach may be especially suitable for patient/sample descriptions where the outcome is affected by interrelated factors that do not need to be analysed separately, e.g. living conditions based on location (respective pollution, violence, hazards, access to healthcare and education in neighbourhoods etc.), occupational hazards and their impact on health, behaviour on social media, money spending habits and many more.

## Conclusions

This study proposes an approach for the construction of data taxonomies to replace descriptive data subsets for data mining and machine learning, and investigates the applicability of taxonomies in classification tasks to substitute raw data or parts of it that do not hold important factors encoded in a single variable. Using *H. pylori* infection as the subject, we have demonstrated that the proposed taxonomy-based approach is valid in correctly identifying groups at a higher risk and in providing possible explanations after comparing groups of people at higher and lower risk. This approach could be used in the future for more complex comparisons, e.g., when analysing the role of human microbiota and the metabolome. This approach is not intended to replace statistical analysis of specific factors and does not serve the same purpose. The taxonomy-based approach can be more convenient than analysing correlations and impacts of individual factors in large and heterogenous data, especially when the attributes can be counted in the hundreds or thousands.

If the specific values of attributes are replaced by a membership to a group and its description, the use of taxonomies can decrease the possibility of identifying individuals in the study. This is a potential direction for future research to improve privacy when sharing anonymized data to promote open science, while improving compactness of the data. The results show that there was insignificant loss of information during this process, and in some cases it can benefit classifier training by reducing overfitting.

## Supplementary Information

**Additional file 1.**

## Data Availability

The data that support the findings of this study are available from the corresponding author on reasonable request. The data are not publicly available due to them containing patient health information and information that could compromise research participant privacy.
