# Supplementary material for: Taxonomy-based data representation for data mining: an example of the magnitude of risk associated with H. pylori infection
Source: BioData Min. 2021 Aug 28;14:43. doi: 10.1186/s13040-021-00271-w (PMC8400764; doi:10.1186/s13040-021-00271-w)
Supplement: Supplementary file 1 — Additional file 1. [file 13040_2021_271_MOESM1_ESM.docx]

Distance measures and linkage methods have a significant impact on the outcome of clustering. There is no rule of thumb to make this decision, therefore it is usually necessary to try several distances and linkages and evaluate them. This should be done using quantitative metrics and qualitative interpretation of the obtained clusters. To illustrate the differences in cluster characteristics, five distance metrics were used for comparison on sociodemographic data subset, which includes the following attributes: sex, marital status, education level, education in years, living arrangement (alone (smaller value, lighter in the heatmap) or with family (larger value, darker in the heatmap)), income level (top to bottom in the heatmaps). The clustering runs included common distance metrics like Manhattan distance and Euclidean distance, as well as metrics used for mixed (continuous, binary and ordinal data) data like the distance proposed by Gower [1] and its improvement proposed by Podani [2], and the distance for mixed data proposed by Ahmad and Dey [3]. These metrics are each used with three linkages: average, centroid and Ward’s linkage. The figures and tables are provided at the end of the table to keep the text easier to read.

One of the quantitative measures that could be helpful in evaluation of clustering methods, distance metrics and linkages is the cophenetic correlation between the distance matrix and the cophenetic distance matrix of the dendrogram, which allows evaluating how well a dendrogram preserves the pairwise distances between the data points [4]. It can also be used to evaluate the cophenetic correlation of dendrograms that have been obtained using different metrics. The number of clusters can be determined using Silhouettes [5] or sums of squares of distances within clusters and between clusters.

When searching for clusters and dendrograms that could be used as taxonomies, there are some specific rules: the clusters have to be different (based on attribute values), which makes them easier to describe, and their sizes should be similar. Therefore sometimes these quantitative metrics do not always provide the best choice for taxonomies. Some of the pitfalls that are most common in this case are:

- A high cophenetic correlation coefficient between the distance matrix and the cophenetic matrix does not always mean that the clusters will be different and therefore provide meaningful descriptions of the groups (or the descriptions will be the same). For example, the best coefficients in Table S1 correspond to Manhattan and Euclidean distances used with average linkage. The heatmap in Fig. S1 shows that there are several clusters different from the mean (lighter and darker shades for clusters 4-6), however, the dendrogram in Fig. S2 shows that these are several small clusters possibly containing significantly different records (clusters 3-6 hold 6 or less records each): divorced less educated women and divorced less educated men with high income level.
- If the clustering process generates small (outlier) clusters, they are not useful as factors contributing to Helicobacter pylori positivity (mostly rare combinations of attribute values). This can be observed in the dendrograms of other distance metrics paired with average or centroid linkage (see Figures S2 and S4). In this case small clusters should be evaluated, and the records could be removed if they are indeed outliers. But this approach would not be optimal if the clusters contain some records with very specific attribute values, which would be clustered differently with different metrics and linkages (e.g. Ward’s linkage in Fig. S6). Therefore, the descriptions of clusters (Heatmaps in Figures S1, S3, S5) should always be analysed in respect to dendrograms. Using different distance metrics provides different results: Manhattan and Euclidean distances provide similar dendrograms, so do Gower’s and Podani’s distances, but the distance proposed by Ahmad and Dey is different (see Tables S2, S3, S4). Therefore, one distance metric of each of the three groups would allow assessing different clusterings.
- The optimal number of clusters specified by a Silhouette width or sums of squares can be suboptimal for a taxonomy. Especially in the case when this number is small (e.g. 2 clusters for sociodemographic data (see Fig. S7), which divides the records into well educated women and less educated men and does not allow for analysis of other factors among women or men). In this case a larger number of clusters has to be selected manually based on cluster descriptions.

**Table S1. Cophenetic correlation coefficient between distance matrixes and cophenetic distance matrixes for average linkage**

| Distance metric | Average linkage | Centroid linkage | Ward’s distance |
| --- | --- | --- | --- |
| Manhattan | 0.8200 | 0.8083 | 0.7305 |
| Euclidean | 0.8203 | 0.7648 | 0.6562 |
| Gower’s | 0.8044 | 0.7979 | 0.7476 |
| Podani’s | 0.8044 | 0.7983 | 0.7407 |
| Ahmad & Day | 0.6597 | 0.6843 | 0.4037 |


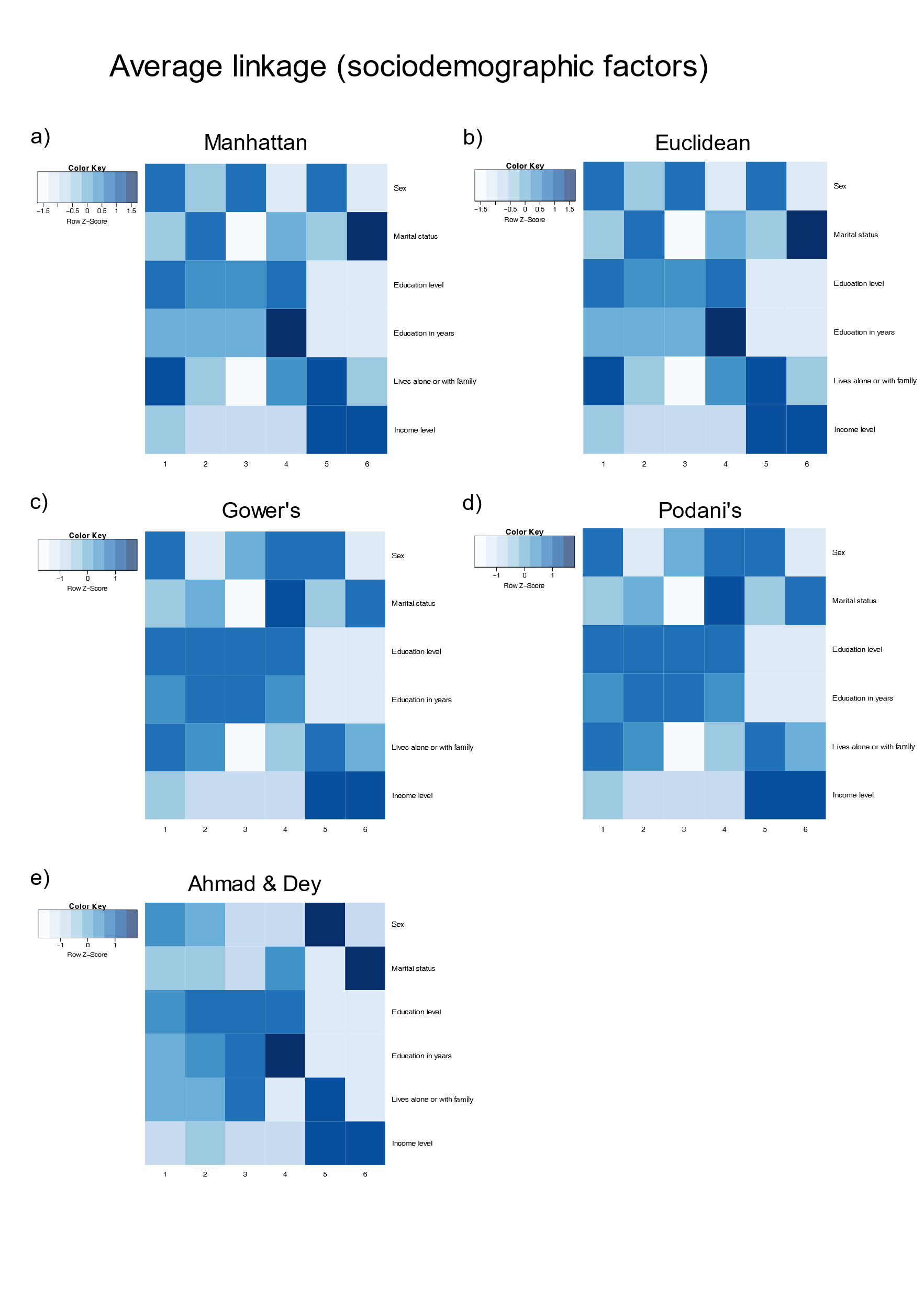


Fig. S1. Heatmaps of sociodemographic factors in the clusters formed using the average linkage and five distance measures


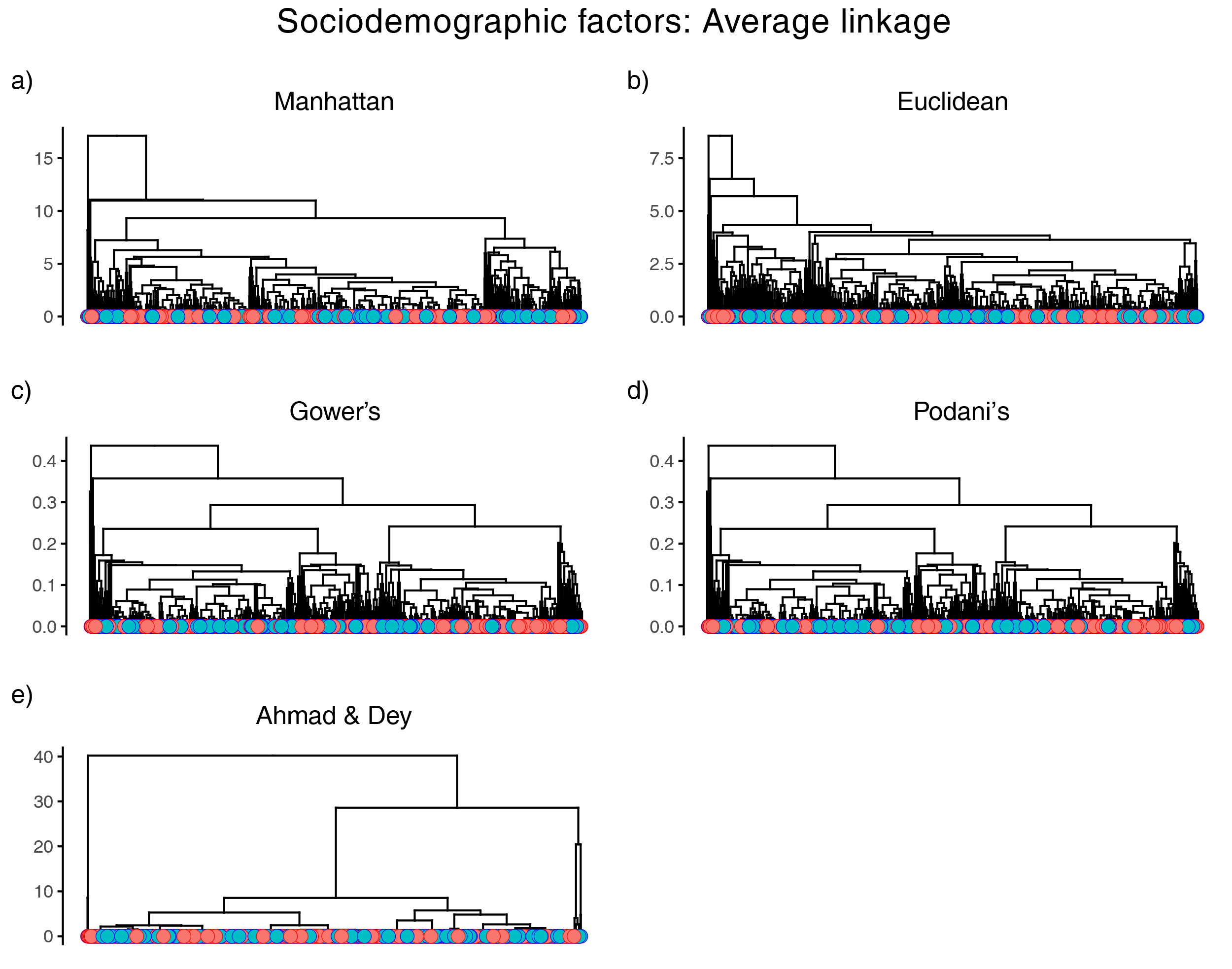


Fig. S2. Dendrogram of the clusters formed using the average linkage and five distance measures

**Table S2. Cophenetic correlation between each two dendrograms calculated using each of the distance metrics and average linkage**

|  | Manhattan | Euclidean | Gower | Podani | Ahmad & Day |
| --- | --- | --- | --- | --- | --- |
| Manhattan | 1.0000 | 0.9543 | 0.4506 | 0.4506 | 0.0191 |
| Euclidean | 0.9543 | 1.0000 | 0.4281 | 0.4290 | 0.0176 |
| Gower | 0.4506 | 0.4281 | 1.0000 | 0.9992 | <0.0001 |
| Podani | 0.4506 | 0.4290 | 0.9992 | 1.0000 | <0.0001 |
| Ahmad & Day | 0.0191 | 0.0176 | <0.0001 | <0.0001 | 1.0000 |


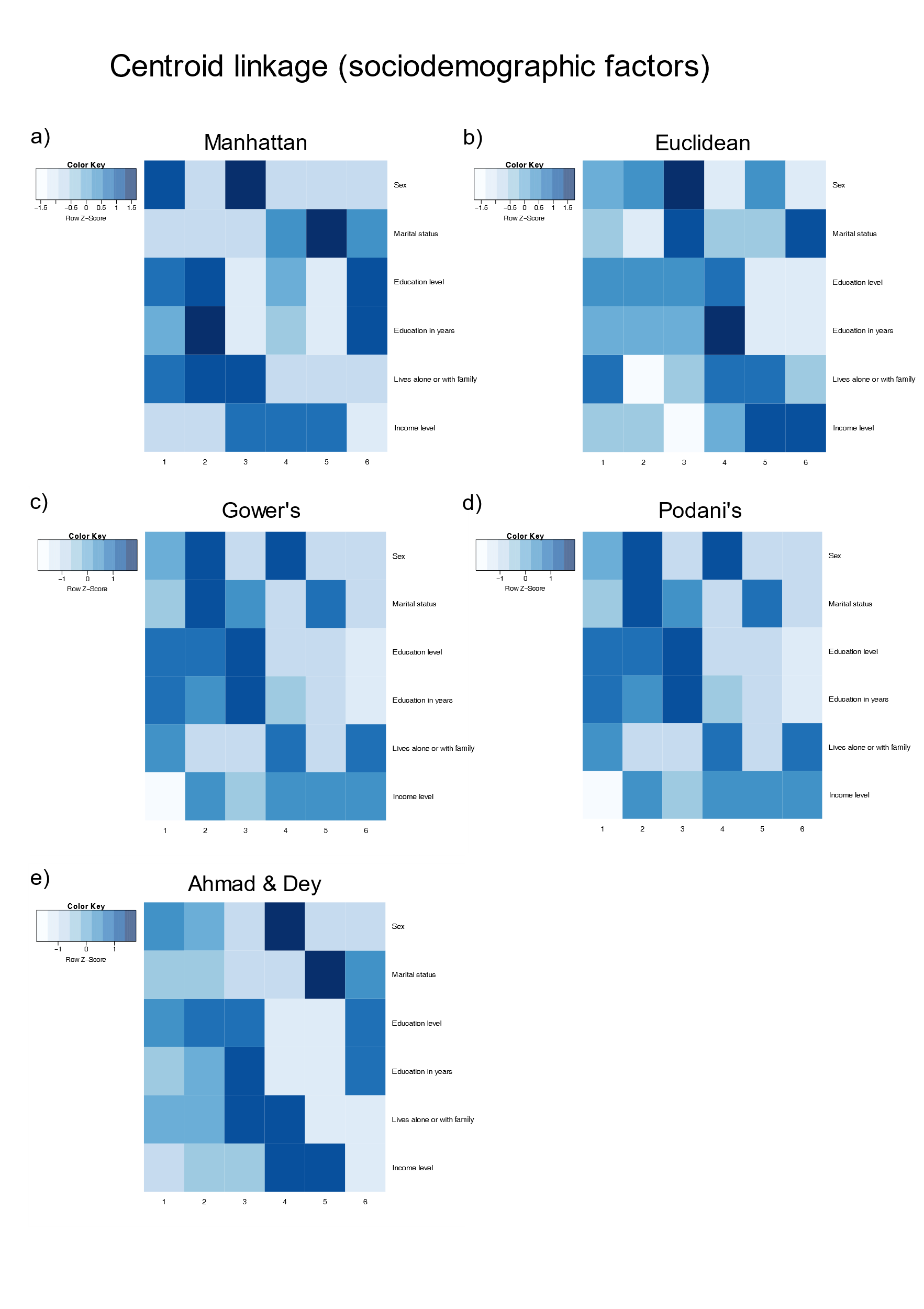


Fig. S3. Heatmaps of sociodemographic factors in the clusters formed using the centroid linkage and five distance measures


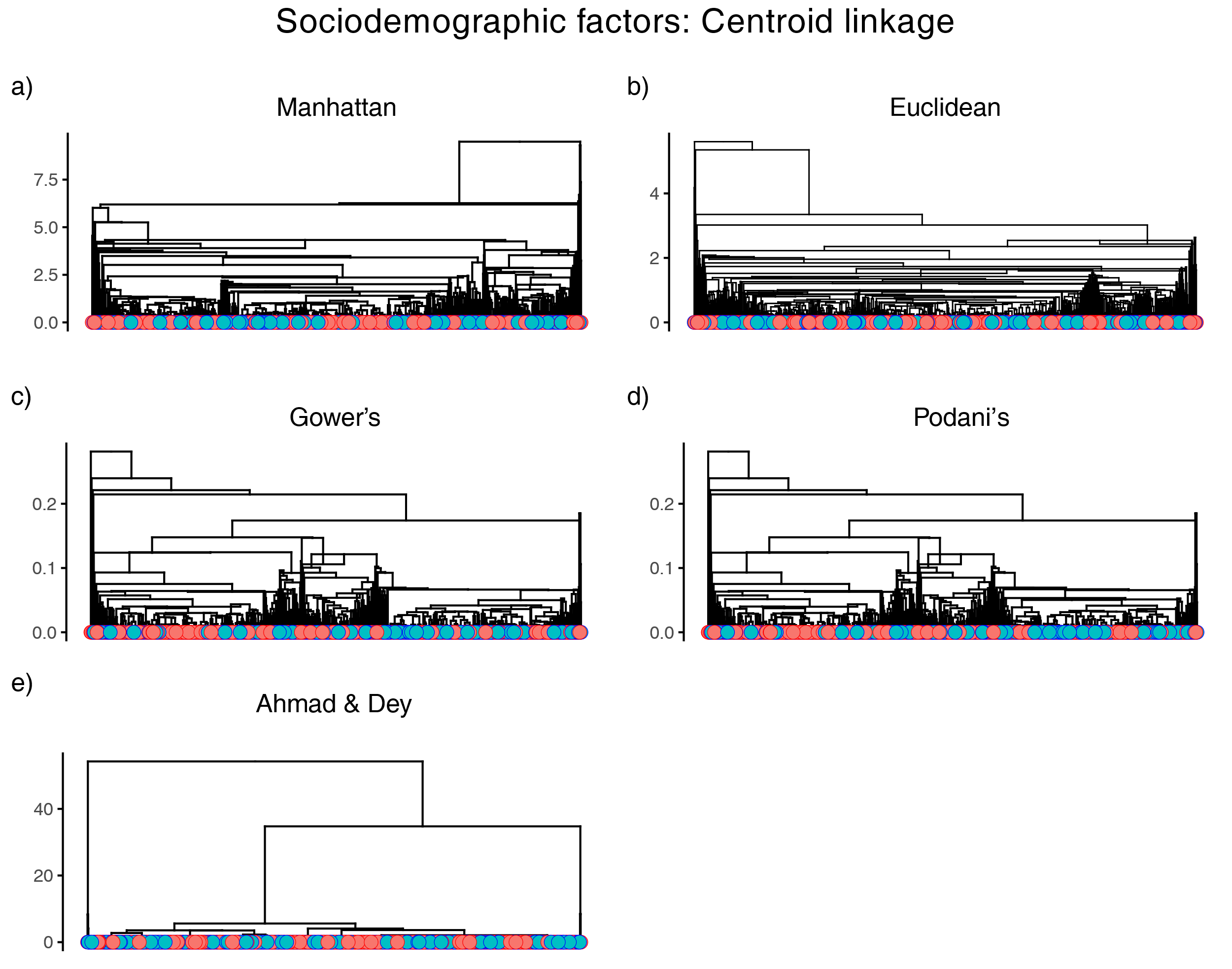


Fig. S4. Dendrogram of the clusters formed using the centroid linkage and five distance measures

**Table S3. Cophenetic correlation between each two dendrograms calculated using each of the distance metrics and centroid linkage**

|  | Manhattan | Euclidean | Gower | Podani | Ahmad & Day |
| --- | --- | --- | --- | --- | --- |
| Manhattan | 1.0000 | 0.8591 | 0.4685 | 0.4686 | -0.0049 |
| Euclidean | 0.8591 | 1.0000 | 0.4128 | 0.4134 | -0.0098 |
| Gower | 0.4685 | 0.4128 | 1.0000 | 0.9990 | -0.0052 |
| Podani | 0.4686 | 0.4134 | 0.9990 | 1.0000 | -0.0046 |
| Ahmad & Day | -0.0049 | -0.0098 | -0.0052 | -0.0046 | 1.0000 |


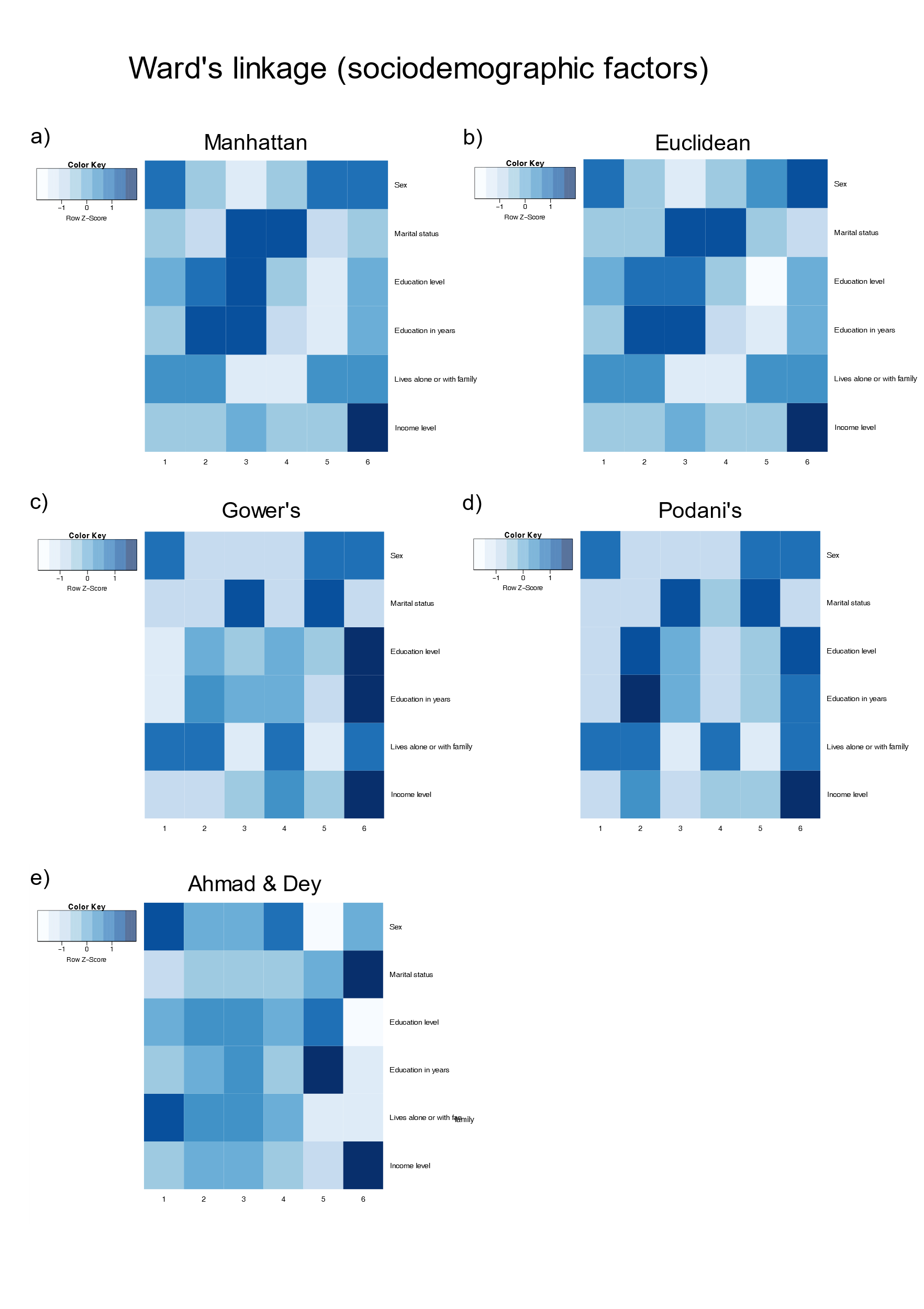


Fig. S5. Heatmaps of sociodemographic factors in the clusters formed using Ward’s linkage and five distance measures


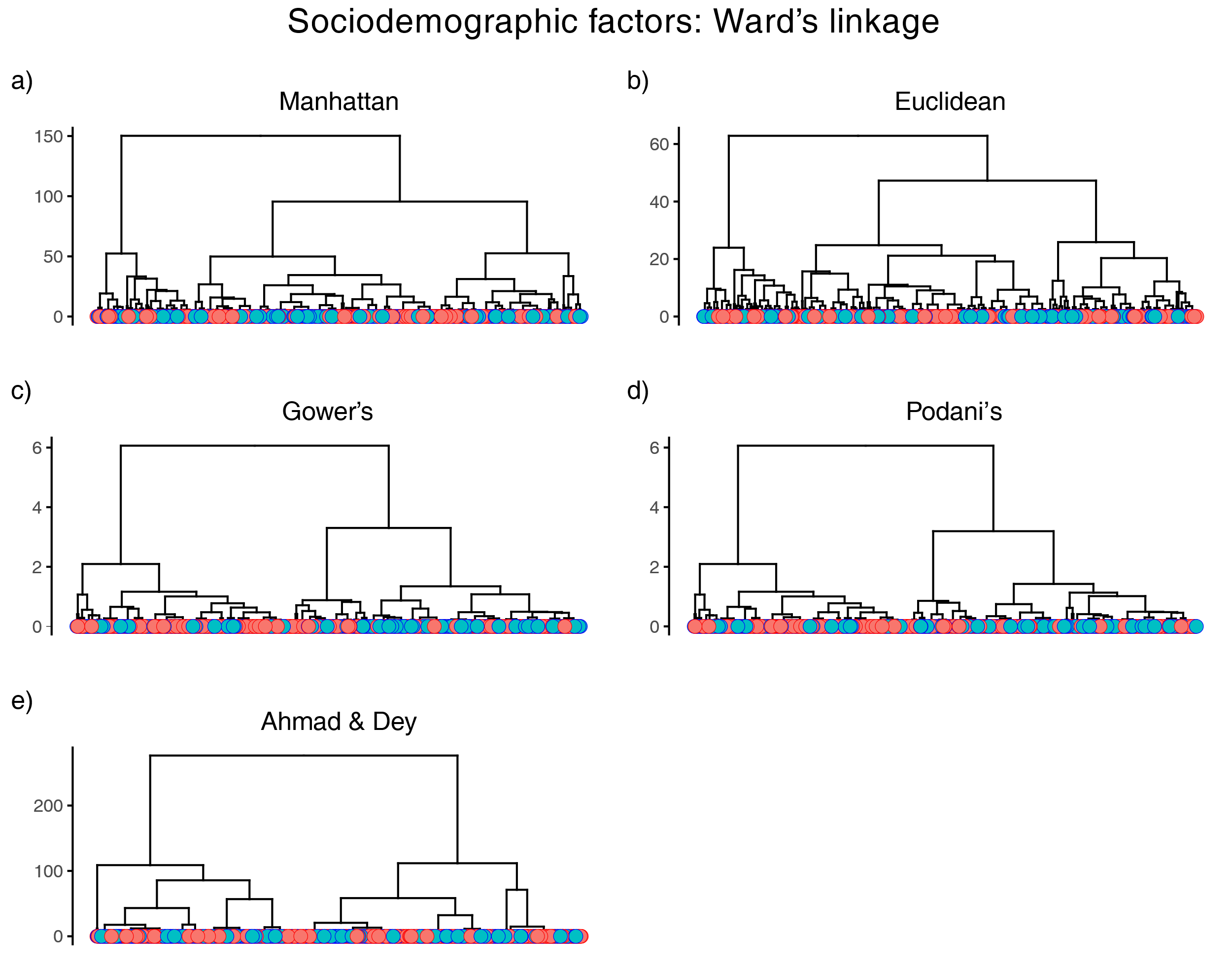


Fig. S6. Dendrogram of the clusters formed using the centroid linkage and five distance measures

**Table S4. Cophenetic correlation between each two dendrograms calculated using each of the distance metrics and Ward’s linkage**

|  | Manhattan | Euclidean | Gower | Podani | Ahmad & Day |
| --- | --- | --- | --- | --- | --- |
| Manhattan | 1.0000 | 0.9597 | 0.2439 | 0.2379 | 0.0020 |
| Euclidean | 0.9597 | 1.0000 | 0.2120 | 0.2082 | 0.0023 |
| Gower | 0.2439 | 0.2120 | 1.0000 | 0.9948 | -0.0010 |
| Podani | 0.2379 | 0.2082 | 0.9948 | 1.0000 | -0.0013 |
| Ahmad & Day | 0.0020 | 0.0023 | -0.0010 | -0.0013 | 1.0000 |


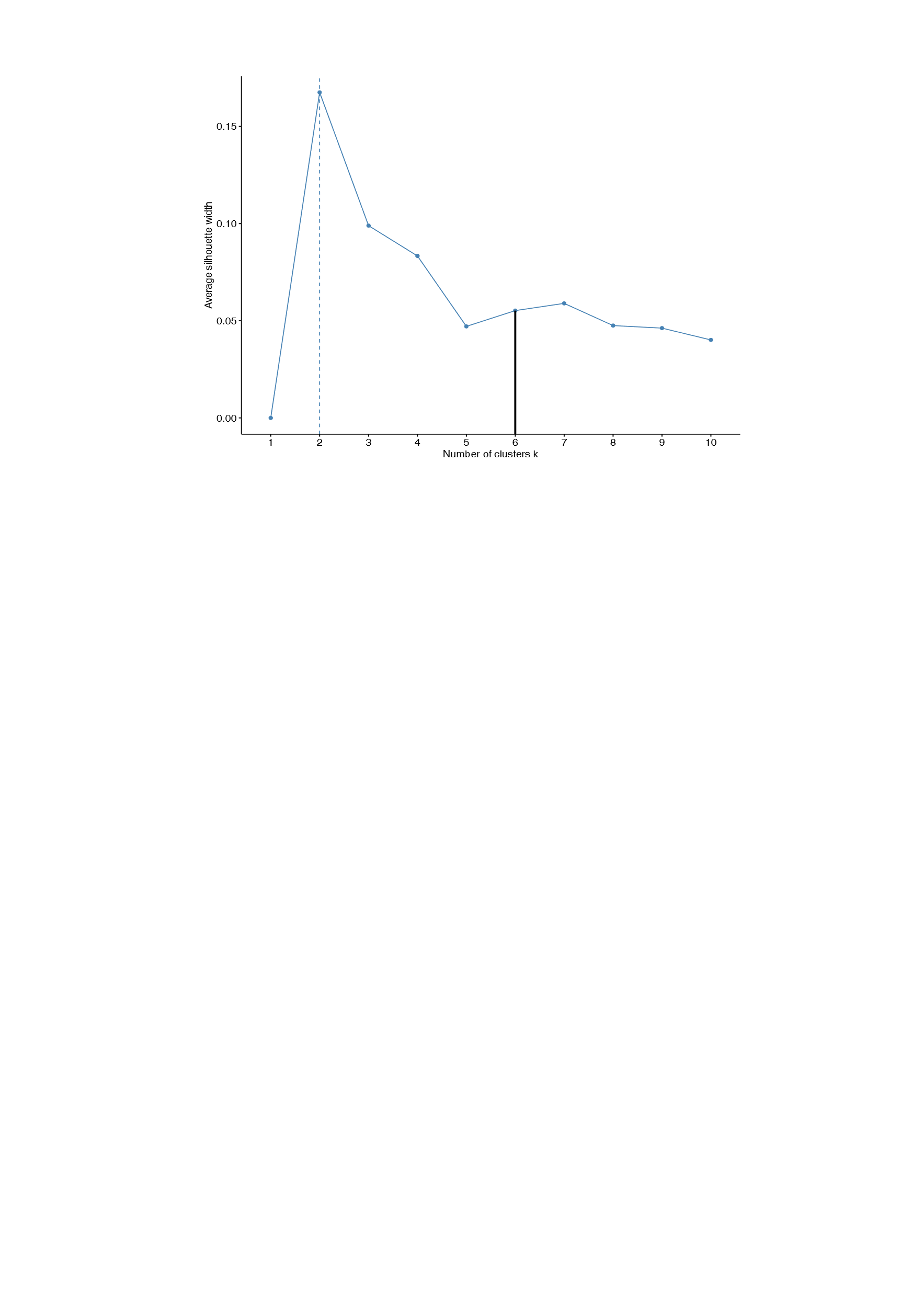


Fig. S7. Silhouette width for 1-10 clusters of the sociodemographic data subset

**References**

1. Gower JC. A General Coefficient of Similarity and Some of Its Properties. Biometrics. 1971;27(4):857–71.

2. Podani J. Extending Gower’s general coefficient of similarity to ordinal characters. Taxon. 1999;48(2):331–40.

3. Ahmad A, Dey L. A k-mean clustering algorithm for mixed numeric and categorical data. Data Knowl Eng. 2007;63(2):503–27.

4. Saraçli S, Doǧan N, Doǧan I. Comparison of hierarchical cluster analysis methods by cophenetic correlation. J Inequalities Appl. 2013;2013(1):203.

5. Rousseeuw PJ. Silhouettes: A graphical aid to the interpretation and validation of cluster analysis. J Comput Appl Math. 1987;20(C):53–65.
